# Healthcare cost consciousness among physicians and their attitudes towards controlling costs in Jordan: a cross sectional study

**DOI:** 10.1186/s12913-022-08834-1

**Published:** 2022-11-25

**Authors:** Mohmmed Hasan Yasin, Abdallah Y. Naser

**Affiliations:** Department of Applied Pharmaceutical Sciences and Clinical Pharmacy, Faculty of Pharmacy, Isra University, Amman, Jordan

**Keywords:** Cost consciousness, Healthcare, Jordan, Physicians

## Abstract

**Background:**

One of the most significant factors influencing medication adherence and, ultimately, therapeutic outcomes for patients is the cost. The aim of this study was to examine the cost-containment strategies used by physicians in Jordan while focusing on the importance of cost consciousness in addressing healthcare costs and its consequences.

**Method:**

A quantitative study was conducted between June 19 and November 15, 2021, through a cross-sectional survey using a self-administered questionnaire.

**Results:**

A total of 389 physicians participated in this study. Governments (65.6%), health insurance companies (60.2%), and pharmaceutical and device manufacturers (57.9%) were the most frequently mentioned entities as being primarily responsible for reducing healthcare costs. Participating physicians showed a high level of enthusiasm towards all domains of reducing healthcare costs with a mean percentage of 88.3% (standard deviation (SD): 0.04). When discussing physicians’ roles in containing healthcare costs and the effects of cost-conscious practice, most respondents agreed that there is currently too much emphasis on test and procedure costs (83.0%), that decision support tools that show costs would be helpful in their practice (84.5%), and that physicians need to take a more prominent role in limiting the use of unnecessary tests (86.0%). Around 70.0% of physicians agreed that they requested more tests when they did not know the patient well, and 80.0% of them stated that they considered the uncertainty involved in patient care to be disconcerting.

**Conclusion:**

Participating physicians showed a moderate level of cost consciousness in Jordan. However, this must be higher because it will eventually lead to cost-related nonadherence, which will have a negative impact on the patient’s health.

## Background

The extent to which a patient is taking medications as prescribed by a physician is typically used to determine compliance with therapy or proper medication use. This entails a number of factors, such as the capacity to obtain medications on prescription, recalling to take medications at the proper dose and time, and understanding the instructions [1]. The rise in healthcare costs is an important barrier to patients receiving treatment. Patients feel they have limited access to the medications and treatments they need to treat their health conditions as medication prices continue to rise. This is seen as a critical issue in healthcare that has a negative impact on patient outcomes [2]. A previous study that assessed the relationship between medication cost and patient adherence showed that an increase in the patient share of medication costs was negatively associated with patient adherence to their therapy. A previous literature review that included 160 articles found that better patient clinical outcomes were associated with higher medication adherence [3]. In a a previous study evaluating cost consciousness among 2556 physicians was carried out in the United States of America (USA) in 2013, physicians demonstrated considerable responsibility for managing practice healthcare costs and generally agreed with a number of quality initiatives to reduce costs. However, they displayed less enthusiasm for cost containment involving modifications to payment methods [4]. Another study in Switzerland in 2005 found that even in a society with very high healthcare costs, physicians’ expressed cost consciousness appeared to be generally high, even though it was not evenly distributed among them [5]. Another study in the USA examined 1000 randomly chosen clinicians who were currently providing direct patient care found that most clinicians believed that only the patient and the treating clinician should make a decision on the cost-effectiveness of a clinical course of action. However, there is disagreement among clinicians over whether to deliver medical interventions with broad potential despite their high cost, and when it comes to cost-effectiveness, their patient interactions are extremely diverse [6]. In a study that examined how the cost of medications affects physicians’ decisions on what medications to prescribe, Hart et al. found that knowing about medication costs can influence a physician’s choice, which eventually lowers medication costs [7]. The best use of healthcare resources is facilitated by physicians who are more knowledgeable about the costs of medications and medical services [8]. One of the most significant factors influencing medication adherence and, ultimately, therapeutic outcomes for patients is cost. According to the latest available statistics in Jordan, around 87.3% of the Jordanian population is covered by health insurance (both private and public health insurance) [9]. Healthcare expenditure accounts for 8.4% of the gross domestic product in Jordan. In 2018, medications costs in Jordan have reached 581 million Jordanian Dinars (818.3 million USD). The aim of this study was to investigate the current state of cost consciousness among Jordanian physicians. Additionally, we sought to investigate the cost-containment strategies used by physicians in Jordan, focusing on their contribution to addressing healthcare costs as well as the consequences of cost consciousness. Understanding cost-conscious attitudes, their prevalence, and all the associated consequences will enable us to offer healthcare organizations and decision-makers suggestions and recommendations that will help to reduce the impact of any negative outcomes related to not being aware of the cost of healthcare.

## Methods

### Study design

A cross-sectional survey study using a self-administered questionnaire was conducted between June 19 and November 15, 2021 to explore the current situation regarding cost consciousness among physicians in Jordan.

### Sampling strategy and sample size

Using a confidence interval of 95%, a standard deviation (SD) of 0.5, a margin of error of 5%, and an 80% power calculation, the minimum required sample size was 385 participants. Practising physicians from all specialties in public and private health facilities who were willing to participate in the study formed the study population. The target sample size was estimated using a population size of 35,000 physicians in Jordan [10]. The following formula was used to estimate the sample size: $\textrm{N}=\frac{Z^2\ast p\hat{\mkern6mu} \left(1-p\hat{\mkern6mu} \right)}{\varepsilon^2}$; z is the z score; ε is the margin of error; N is the population size; p̂ is the population proportion.

### Sampling procedure

A convenience sampling technique was used to recruit eligible participants. This sampling technique is a type of non-probability sampling where participants from the target population are included if they match the research’s inclusion criteria and are easily reachable due to proximity to the study site, availability at a specific time, or willingness to participate.

### Participant recruitment

Physicians working in Jordan, whether in the private sector or the public sector, as residents or specialists, and who have expressed a desire to participate in the study, form the study population. Practicing physicians from all specialties and levels of decision-making in Jordan were approached and invited to participate in the study using an online and paper-based survey tool. The online version was sent to them through relevant (medical groups and pages) social media platforms, including Facebook and WhatsApp. In addition, using Qualtrics survey software, the survey link was circulated to all physicians working at governmental and private hospitals in Jordan after contacting the management of these healthcare centres by the researcher. Two weeks after the initial invitation email was sent, the participants received a reminder email. Paper surveys were also provided by visiting the physicians in their clinics. The paper-based survey was changed for individuals who wanted the electronic format by sending the questionnaire to the physician’s email address. After outlining the aim and objectives of the study, physicians who consented to participate were given the link or the paper-based draft of the questionnaire. For further explanation of the study, participants were provided information sheets. Additionally, they were informed that by responding to the questionnaire, they were giving their written consent to participate in the study. Finally, they were informed that participation in the study is completely voluntary and that they are free to withdraw at any time without having to give a reason.

### The questionnaire tool

A previously validated questionnaire developed by Tilburt et al. was used in this study [4]. The questionnaire used in this study was written in English. The questionnaire consisted of 5 sections. The first section asked the participants about their demographic and practice characteristics. The second section explored physicians’ and society’s responsibilities to reduce the cost of healthcare. The participants were given nine options (pharmaceutical and device manufacturers, hospitals and health systems, individual practising physicians, the government, health insurance companies, professional societies, trial lawyers, employers, and patients) to determine which 1 was more responsible. The responses that participants could select regarding the degree of responsibility were “no responsibility”, “some responsibility”, and “major responsibility”. The third section assessed physicians’ enthusiasm for reducing healthcare costs through 18 items representing 4 domains: improving quality and efficiency of care (6 items), improving conditions for evidence-based decisions (3 items), changing how care gets paid for (6 items), and cutting payment to physicians directly (3 items). The responses of participants regarding their degree of enthusiasm could be “not enthusiastic”, “somewhat enthusiastic”, and “very enthusiastic”. The fourth section explored physicians’ role in containing healthcare costs and the consequences of cost-conscious practices. The responses of participants were recorded using a 4-point Likert scale that ranged from “strongly disagree” (given a score of 1) to “strongly agree” (given a score of 4). To identify the predictors of cost consciousness (highlighted in Table 3 with the symbol “*”), only 11 items were included in the derivation of the cost-consciousness score. On this scale, a score of 44 was the highest possible score. The last section asked the participants 2 items about the consequences of cost-conscious practice.

### Survey piloting

A pilot study was conducted on 30 physicians who had met the inclusion criteria to confirm their understanding of the questionnaire and whether it was measuring what we were aiming to measure. Physicians who participated in the piloting phase were working in the private and public sectors and were chosen to represent different specialties to match the study’s targeted population. The physicians confirmed the content and face validity of the questionnaire and found it clear and easy to measure cost consciousness among Jordanian physicians.

### Statistical analysis

The data was analyzed using the SPSS software, version 27. The descriptive analysis was reported as mean (μ) ± SD. Descriptive statistics were used to describe the participants’ demographic information. The normality of the data was checked using the histogram and normality measures (skewness and kurtosis were checked). Categorical data were reported in the form of percentages and frequencies. The mean cost-consciousness score was estimated using a 4-point Likert scale that ranged between strongly disagree “score 1″ to strongly agree “score 4″ with a maximum score of 44 (11-items). Binary logistic regression was used to determine factors that are associated with being cost-conscious. The mean cost-consciousness score (28.0 (SD: 2.4) out of 44) was the cut-off value used to define the dummy variable for the logistic regression analysis. The assumption of multicollinearity across all independent variables was checked using correlation analysis. A confidence interval (CI) of 95% (*p* < 0.05) was applied to represent the statistical significance of the results, and the level of significance was assigned as 5%.

## Results

### Physicians’ demographic and practice characteristics

A total of 389 physicians participated in this study, of whom 60.9% were males. Around half of the study participants (51.2%) were aged 40 years or less. Almost one-third of the study participants (29.0%) reported that they had a total experience of 5 years or less. More than half the study participants (57.8%) were general practitioners who had completed their first degree in medicine in Jordan. Dermatologists comprised 10.0% of the total number of participants. The vast majority (94.5%) of specialized physicians reported that they had completed their specialty education in Jordan.

Regarding practice sites, only 26.7% of the participating physicians reported that they work only in the public healthcare sector, while the remaining physicians reported that they work either in the private healthcare sector or in both sectors. When the participating physicians were asked about the compensation methods they accepted for healthcare practices, the majority (78.4%) reported that they accepted both direct payment and patients covered by health insurance. Around half the study participants (50.6%) reported that their location of practice was Amman, the capital of Jordan. For further details on the demographic and practice characteristics of participating physicians, please refer to Table 1.

**Table 1:** Demographic and practice characteristics of participating physicians

Demographic variable	Frequency	Percentage
Gender		
Males	237	60.9%
Females	152	39.1%
Age category		
23-30 years	98	25.2%
31-35 years	59	15.2%
36-40 years	42	10.8%
41-45 years	35	9.0%
46-50 years	41	10.5%
51 years and above	114	29.3%
Years of experience		
Less than 5 years	113	29.0%
6 to 10 years	100	25.7%
More than 10 years	176	45.2%
Medical education		
General practitioner	223	57.8%
Specialized physician	163	42.2%
Country of education (first medical degree)		
Jordan	239	61.4%
Outside Jordan	150	38.6%
Specialty		
Resident	14	3.6%
Internist	12	3.1%
Surgeon	17	4.4%
Gynecologist	28	7.2%
Pediatrician	17	4.4%
Dermatologist	39	10.0%
Orthopedic	12	3.1%
ENT	9	2.3%
Other specialty	15	3.9%
Country of education (for specialized physicians) (*n* = 163)		
Jordan	154	94.5%
Outside Jordan	9	5.5%
Practice site		
Public healthcare sector	104	26.7%
Private healthcare sector	135	34.7%
Both	150	38.6%
Compensation method accepted for healthcare practices:		
Direct payment only	66	17.0%
Patients covered by health insurance only	18	4.6%
Both methods	305	78.4%
Location of practice:		
Amman	197	50.6%
Aqaba	15	3.9%
Madaba	14	3.6%
M’aan	7	1.8%
Balqa	23	5.9%
Jarash	14	3.6%
Ajloun	7	1.8%
Tafelah	17	4.4%
Karak	21	5.4%
Zarqa	35	9.0%
Mafraq	12	3.1%
Irbid	27	6.9%

### Physicians’ and society’s responsibility to reduce the cost of healthcare

**Fig. 1:**
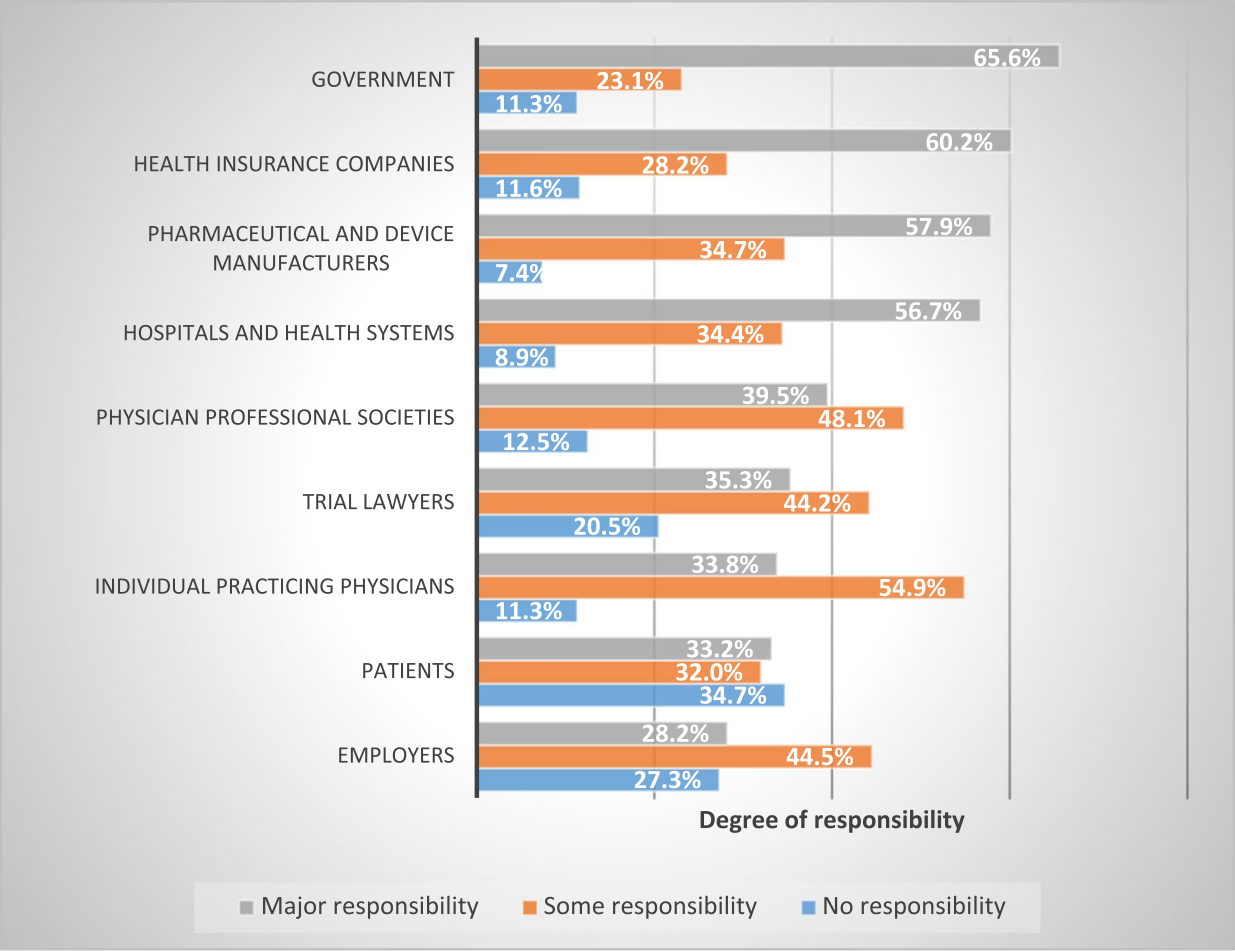
Degree of responsibility of physicians and society to reduce the cost of healthcare

When the participating physicians in this study were asked about whose responsibility it was to reduce the cost of healthcare, the most frequently mentioned bodies that have the main responsibility for reducing the cost of healthcare were the governments (65.6%), health insurance companies (60.2%), and pharmaceutical and device manufacturers (57.9%). For further details on the degree of agreement regarding physicians’ and society’s responsibilities to reduce the cost of healthcare, please refer to Fig. 1.

### Enthusiasm for reducing healthcare costs

Physicians’ enthusiasm for reducing healthcare costs was assessed through 18 items representing 4 domains: improving quality and efficiency of care, improving conditions for evidence-based decisions, changing how care gets paid for, and cutting payment to physicians directly, as presented in Table 2.

**Table 2:** Physicians’ enthusiasm for reducing healthcare costs

	Degree of enthusiasm (%)		
	Not enthusiastic	Somewhat enthusiastic	Very enthusiastic
Improving quality and efficiency of care:			
Promoting continuity of care	25 (6.4%)	199 (51.1%)	166 (42.6%)
Expanding electronic health records	39 (9.9%)	185 (47.5%)	166 (42.6%)
Promoting better conversations/communication with patients	37 (9.6%)	186 (47.9%)	166 (42.6%)
Promoting chronic disease care coordination	30 (7.8%)	204 (52.5%)	154 (39.7%)
Rooting out fraud and abuse	47 (12.1%)	189 (48.6%)	153 (39.4%)
Expanding access to free preventive care	36 (9.2%)	219 (56.4%)	134 (34.4%)
Improving conditions for evidence-based decisions:			
Expanding access to quality and safety data	35 (8.9%)	189 (48.6%)	166 (42.6%)
Promoting head-to-head trials of competing treatments	41 (10.6%)	205 (52.8%)	142 (36.5%)
Limiting corporate influence on physician behavior	53 (13.5%)	204 (52.5%)	132 (34.0%)
Changing how care gets paid for:			
Using cost-effectiveness data to determine available treatments	39 (9.9%)	185 (47.5%)	166 (42.6%)
High-deductible health plans	30 (7.8%)	212 (54.6%)	146 (37.6%)
Limiting access to expensive treatments with little net benefit	43 (11.0%)	207 (53.2%)	139 (35.8%)
Penalizing providers for avoidable readmissions	65 (16.7%)	195 (50.0%)	130 (33.3%)
Higher patient co-payment	55 (14.2%)	222 (57.1%)	112 (28.7%)
Paying a network of practices, a fixed, bundled price for managing all care for a defined population	47 (12.1%)	232 (59.6%)	110 (28.4%)
Cutting payment to physicians directly:			
Reducing compensation for the highest-paid specialties	51 (13.1%)	223 (57.4%	114 (29.4%
Eliminating fee-for-service payment models	84 (21.6%)	202 (51.8%	103 (26.6%
Allowing health insurance payment cuts to doctors to take effect	66 (17.0%)	228 (58.5%	95 (24.5%

Overall, participating physicians showed a high level of enthusiasm towards all domains of reducing healthcare costs with an enthusiasm percentage (from “somewhat enthusiastic” to “very enthusiastic”) of a mean percentage of 88.3% (SD: 0.04). The mean enthusiasm percentage for the 4 domains was relatively high and ranged between 82.7 and 90.9%. The highest enthusiasm percentage was for the “improving the quality and efficiency of care” domain, which was 90.9% (SD: 0.02), while the lowest enthusiasm percentage was for the “cutting payments to physicians directly” domain, which was 82.7% (SD: 0.04). The remaining 2 domains, “improved conditions for evidence-based decisions” and “change how care gets paid for” domains, were agreed upon by 89.0% (SD: 0.02) and 88.1% (SD: 0.03), respectively.

### Physicians’ role in containing healthcare costs and the consequences of cost-conscious practice

The physicians’ role in containing healthcare costs and the consequences of cost-conscious practice were assessed using 13 items. The most commonly agreed-upon items were: “there is currently too much emphasis on the costs of tests and procedures” (83.0%), “decision support tools that show costs would be helpful in my practice” (84.5%), and “physicians need to take a more prominent role in limiting the use of unnecessary tests” (86.0%). For further details on physicians’ role in containing healthcare costs and the consequences of cost-conscious practice, please refer to Table 3.

**Table 3:** Physicians’ attention and agreement regarding their role in containing health care costs

	Degree of agreement (%)			
	Strongly disagree	Moderately disagree	Moderately agree	Strongly agree
I am aware of the costs of the tests/treatments I recommend	16 (4.1%)	55 (14.2%)	277 (71.3%)	40 (10.3%)
I try not to think about the cost to the health care system when making treatment decisions^a^	25 (6.5%)	248 (63.8%)	77 (19.9%)	38 (9.8%)
I should sometimes deny beneficial but costly services to certain patients because resources should go to other patients that need them more	31 (8.0%)	72 (18.6%)	267 (68.7%)	18 (4.7%)
Cost to society is important in my decisions to use or not to use an intervention^a^	10 (2.6%)	68 (17.6%)	291 (74.7%)	20 (5.2%)
Physicians should adhere to clinical guidelines that discourage the use of interventions that have a small proven advantage over standard interventions but cost much more*	17 (4.4%)	53 (13.7%)	284 (72.9%)	35 (9.0%)
The cost of a test or medication is only important if the patient has to pay for it out of pocket^a^	34 (8.8%)	267 (68.7%)	62 (16.0%)	25 (6.5%)
Doctors are too busy to worry about costs of tests and procedures^a^	23 (5.9%)	272 (70.0%)	65 (16.8%)	28 (7.2%)
Trying to contain costs is the responsibility of every physician^a^	12 (3.1%)	64 (16.5%)	286 (73.4%)	27 (7.0%)
There is currently too much emphasis on costs of tests and procedures^a^	10 (2.6%)	56 (14.5%)	296 (76.0%)	27 (7.0%)
Doctors need to take a more prominent role in limiting use of unnecessary tests^a^	7 (1.8%)	47 (12.1%)	284 (73.1%)	50 (12.9%)
It is unfair to ask physicians to be cost-conscious and keep the welfare of their patients foremost in their minds^a^	18 (4.7%)	294 (75.5%)	62 (16.0%)	15 (3.9%)
I should be solely devoted to my individual patients’ best interests, even if that is expensive^a^	28 (7.2%)	275 (70.8%)	63 (16.3%)	22 (5.7%)
Decision support tools that show costs would be helpful in my practice^a^	11 (2.8%)	49 (12.7%)	286 (73.4%)	43 (11.1%)

^a^Included in the 11-item cost consciousness scale

### The consequences of cost-conscious practice

The consequences of cost-conscious practice were assessed using 2 items. When the participating physicians were asked whether they found the uncertainty involved in patient care disconcerting, more than 80.0% reported their agreement. When the participating physicians were asked whether they ordered more tests when they did not know the patient well, around 70.0% reported their agreement (please see Table 4).

**Table 4:** Consequences of cost-conscious practice

	Degree of agreement (%)			
	Strongly disagree	Moderately disagree	Moderately agree	Strongly agree
I find the uncertainty involved in patient care disconcerting “worrying”:	16 (4.1%)	58 (15.0%)	217 (55.9%)	97 (25.0%)
I generally order more tests when I don’t know the patient well:	70 (18.1%)	46 (11.9%)	83 (21.3%)	190 (48.8%)

### Predictors of physicians’ cost consciousness

The mean cost-consciousness score was 28.0 (SD: 2.4) out of 44, the maximum obtainable score (which is equal to 63.6%) and ranged between 21 and 41. Physicians who were aged 31–35 years and had 6 to 10 years of experience were more likely to be cost-conscious compared to others with (OR: 3.01(95% CI: 1.16–7.81)) and (OR: 3.00 (95% CI: 1.12–7.80)). On the other hand, older physicians (aged 51 years and above) and those who had received their first medical degree education outside Jordan were less likely to be cost-conscious compared to others (OR: 0.47 (95% CI: 0.28-0.78)) and (OR: 0.58 (95% CI: 0.35-0.95)), respectively, see Table 5.

**Table 5:** Predictors of physicians’ cost consciousness

Demographic variable	Crude Odds ratio	95% CI	*P*-value
Gender			
Males (Reference group)	1.00		
Females	1.24	(0.74-2.08)	0.421
Age category			
23-30 years (Reference group)	1.00		
31-35 years	**3.01**	**(1.16-7.81)**	0.023*
36-40 years	1.06	(0.47-2.38)	0.898
41-45 years	1.21	(0.49-3.03)	0.680
46-50 years	1.02	(0.45-2.31)	0.962
51 years and above	**0.47**	**(0.28-0.78)**	0.004**
Years of experience			
Less than 5 years (Reference group)	1.00		
6 to 10 years	**3.00**	**(1.12-7.80)**	0.024*
More than 10 year	1.06	(0.47-2.32)	0.899
Medical education			
General practitioner (Reference group)	1.00		
Specialized physician	1.11	(0.67-1.84)	0.696
Country of education (first medical degree)			
Jordan (Reference group)	1.00		
Outside Jordan	**0.58**	**(0.35-0.95)**	0.031*
Country of specialized education (for specialized physicians)			
Jordan (Reference group)	1.00		
Outside Jordan	0.84	(0.44-1.61)	0.598
Practice site			
Public healthcare sector (Reference group)	1.00		
Private healthcare sector	1.22	(0.71-2.08)	0.467
Both	0.800	(0.48-1.33)	0.388
Compensation method accepted for healthcare practices:			
Direct payment only (Reference group)	1.00		
Patients covered by health insurance only	0.63	(0.22-1.81)	0.388
Both methods	0.85	(0.46-1.59)	0.615
Location of practice:			
Amman (Reference group)	1.00		
Other cities	1.30	(0.79-2.14)	0.310

*p ≤ 0.05; **p ≤ 0.01

## Discussion

This study explored the current situation regarding cost consciousness among physicians in Jordan. Additionally, it explored cost containment strategies applied by physicians in Jordan, paying attention to their role in addressing healthcare costs and the consequences of cost consciousness. The key findings are: 1) the most commonly reported bodies to have the major responsibility for reducing the cost of healthcare were the governments, health insurance companies, and pharmaceutical and device manufacturers; 2) participating physicians showed enthusiasm towards all domains of reducing healthcare costs; 3) more than 80.0% reported that uncertainty involved in patient care is disconcerting, and 70.0% reported that they order more tests when they do not know the patient well; 4) participants showed a moderate level of cost consciousness (63.6%); 5) early age (31-35 years) and having 6 to 10 years of experience were important predictors of having a high level of cost consciousness, while older age (51 years and above) and receiving their first medical degree education outside Jordan were important predictors of having a low level of cost consciousness.

Jordanian physicians’ opinions on their role in containing healthcare costs are complex. In this survey, we found that the most commonly reported bodies to have the major responsibility for reducing the cost of healthcare from the physicians’ perspective were the governments, health insurance companies, and pharmaceutical and device manufacturers. In comparison with previous studies [4, 11], it was found that physicians think the most frequently reported institutions are primarily responsible for reducing medical costs are health insurance companies, lawyers, hospitals, and healthcare systems. As for the party responsible for reducing the price of medicine, most of the physicians in the current study in Jordan agree with a previous study that the responsible authorities are the same, including the governments, health insurance companies, and pharmaceutical and device manufacturers [4]. Jordan charges a 4% tax on all pharmaceutical products, medical devices, and consumables [12]. This increases the overall cost of healthcare provision on healthcare providers themselves and, ultimately, on the patients. Apart from these general conclusions, Jordanian physicians recognized that commitments related to cost management are complicated. Their opinions in the survey are related to the place and work environment, whether in the public or private clinics, the type of specialization, and the method of payment. Health insurances in Western societies cover patients better in all aspects of healthcare, while in our societies these insurances cover part of the cost of healthcare and there are many healthcare services and medications are not covered in these insurances [13]. Therefore, we found that physicians in a previous study were in agreement that health insurance companies had more than 95% of the responsibility for lowering the cost of healthcare [4].

In Tilburt et al.’s study, 70% of physicians were “not enthusiastic” about reducing fee-for-service [4]. The results were similar in Jordan in this regard, as only around 25% were “very enthusiastic”, and 75% of the remaining physicians were either ‘not enthusiastic at all’ or “somewhat enthusiastic”. Although the income of an American physician is higher than that of a Jordanian physician, the problem exists in Jordan as well [14], and we certainly expected this result, especially since the physician’s income, as we mentioned, is lower in Jordan. The reason is for this result is typically related to the fact that physicians in Jordan finds that their income is relatively low compared to physicians in other countries.

Overall, participating physicians showed a high level of enthusiasm towards all domains (being “somewhat enthusiastic” to “very enthusiastic”), with a mean enthusiasm percentage of 88.3% (SD: 0.04). They were more enthusiastic about improving “the quality and efficiency of care”, followed by “improving conditions for evidence-based decisions”. This high percentage) 88.3% (indicates the Jordanian physicians’ awareness of the need to change for the better. This percentage compared to a previous study is close, as the American physicians showed a very high rate of enthusiasm, over 95%, about ‘promoting continuity of care’, despite the high level of medical care in the United States compared to Jordan, in addition to the availability of medical equipment and the better financial level for American physicians [4].

More than 80.0% of the participating physicians reported that they find the uncertainty involved in patient care disconcerting. A previous study in the United States found that 78% of American physicians who participated in the survey also agreed that “[their] individual patients should be devoted to the individual patients’ best treatment despite the high prices” [4]. When the physician is not fully aware of the prices or the status of his patient’s health insurance or the general financial situation of the patient, this makes the physician unable to balance matters by prescribing the optimal treatment for the patient and at the same time maintaining the financial situation and not adding large costs to the patient, who also has to pay the physician’s fee in addition to paying for medical tests in the medical laboratory and the prescribed medicines and other medical costs. In this case, the physician is uncertain because he or she wants to prescribe the best treatment for the patient but, at the same time, does not want to add large costs to the patient. A previous study in Jordan reported that the prevalence of cost-related nonadherence in Jordan is around 30.0%, and it was most common in patients with hypertension, diabetes, low incomes, and low levels of education [15].

When the Jordanian physicians were asked about cost containment and their role in containing healthcare costs around 85% of physicians showed their agreement to the statement that physicians need to take a more prominent role in limiting the use of unnecessary test. Conversely, around 70.0% of the participating physicians reported that they order more tests when they do not know the patient well. When comparing this result with the American physicians, we fond that 42% of American physicians “strongly or moderately agree”, and 58% “moderately or strongly disagree” that they do not order more tests when they do not know the patient well [4]. This result is somewhat logical, as physicians in Jordan usually request many laboratory tests, which are expensive for the patient. In addition, the reason is that, unlike in the USA, there is no electronic healthcare system that includes all patients’ clinical information in Jordan [16], and this requires the physician to ask for a higher number of tests to properly diagnose the patient. Despite the fact that we found multiple similarities in terms of physicians’ perception regarding their responsibility to reduce the cost of healthcare, their enthusiasm for reducing healthcare costs, and their role in containing healthcare costs and consequences of cost-conscious practice, it’s worth mentioning that healthcare structure and access to healthcare are different between Jordan and the USA.

Participating physicians showed a moderate level of cost consciousness with a mean score of 28.0 (SD: 2.4) out of 44, the maximum obtainable score (which is equal to 63.6%). Deep drilling and careful study of cost consciousness will have a positive impact on the patient’s life. Physicians’ awareness of healthcare costs is an important matter that includes healthcare providers’ knowledge of all matters related to the healthcare system as a whole, whether it is the cost of medications or laboratory tests, the cost of medical devices, or knowledge of the details of patients’ health insurance policies [6, 17]. By knowing these important details about healthcare costs, physicians can give the most cost-effective treatment, which will reflect positively on the patient’s health condition. If the cost to the patient remains high, this will lead to the problem of cost-related non-adherence [15].

Physicians who were aged 31–35 years and had 6 to 10 years of experience were more likely to be cost-conscious compared to others with (OR: 3.01(95% CI: 1.16–7.81)) and (OR: 3.00 (95% CI: 1.12–7.80)). On the other hand, older physicians (aged 51 years and above) and those who had received their first medical degree education outside Jordan were less likely to be cost-conscious compared to others (OR: 0.47 (95% CI: 0.28-0.78)) and (OR: 0.58 (95% CI: 0.35-0.95)), respectively. It was found in our study that young physicians were more cost-conscious as they had recently finished their study and residency programmes, which gave them updated and detailed information about medication prices and other information, so their information about Jordan medication prices was better. In addition, they were enthusiastic as they were at the beginning of their practice of medicine, and it was important for them not to raise costs on their patients and to treat them at the lowest cost in order to earn more in the future and to improve their reputation in the medical community.

In our study, we found that participating physicians showed a moderate level of cost consciousness; nevertheless, this level must be higher because it will ultimately result in cost-related nonadherence, which will have a negative impact on the health of the patient [15]. A previous study in Jordan explored cost considerations of dermatological care reported that 27.7% of the patients said their dermatologist does not go over the cost of medications with them. Additionally, about 71.4% of respondents stated that getting out-of-pocket medication expenditure estimates is crucial for them [18].

Pharmaceutical companies and healthcare organizations in Jordan should raise physicians’ awareness of costs so they can use medications most effectively and promote cost-effectiveness. This ultimately leads to better patient health outcomes as it will reduce cost-related non-adherence. These efforts should be directed mainly towards elderly physicians and those who studied outside Jordan. Pharmaceutical companies should focus on these physicians when making visits, in addition to conducting educational workshops and conferences where these physicians should be targeted.

The level of awareness among physicians must be increased by holding continuous conferences and educational seminars on the application of treatment to patients and the definition of the price of medications and their updates [19]. Decision makers in the healthcare sector, in addition to pharmaceutical companies, are advised to raise healthcare professionals’ cost-consciousness. We recommend that future studies should focus on exploring cost-consciousness in different disease areas, whether chronic or acute, to implement the appropriate cost-effective intervention, whether through medical conferences, continuing education, or instructions through the Ministry of Health.

To the best of our knowledge, this is the first study to assess the current situation regarding cost consciousness among physicians in Jordan. Our study cohort was not limited to certain specialties or practice settings (private or governmental), which will improve the generalizability of our findings. However, this study has limitations. The physicians were given online or paper-based surveys as part of a cross-sectional study design. Social desirability bias may be present in a self-administered questionnaire. As a non-probability sampling method was used in this study, the use of convenience sampling may have had an impact on how generalizable our findings were. We were unable to compare our findings with those of Middle Eastern countries with a similar healthcare environment and culture since no previous research on physicians’ cost-consciousness had been conducted in the region. Therefore, our findings should be interpreted carefully.

## Conclusion

A moderate level of cost consciousness was found among practicing physicians in Jordan. We recommend increasing cost consciousness and doing additional research into other reasons for the lack of cost consciousness. Physicians’ awareness of healthcare prices and costs will directly reflect a better health life for patients, in addition to reducing and directing better financial spending from the government.

## Data Availability

All data generated or analysed during this study are included in this published article.

## Abbreviations

USA: United States of America
SPSS: Statistical Package for Social Sciences
SD: Standard Deviation
